# Prevalence and factors associated with depressive symptoms among post-partum mothers in Nepal

**DOI:** 10.1186/s13104-015-1074-3

**Published:** 2015-03-31

**Authors:** Rajendra Kumar Giri, Resham Bahadur Khatri, Shiva Raj Mishra, Vishnu Khanal, Vidya Dev Sharma, Ritu Prasad Gartoula

**Affiliations:** Department of Ayurveda, Ministry of Health and Population, Kathmandu, Nepal; Save the Children, Saving Newborn Lives Program, Kathmandu, Nepal; Nepal Development Society, Kathmandu, Nepal; School of Public Health, Curtin University, Perth, Australia; Department of Psychiatry, Institute of Medicine, Tribhuwan University, Kathmandu, Nepal; Department of Community Medicine and Public Health, Institute of Medicine, Tribhuwan University, Kathmandu, Nepal

**Keywords:** Edinburgh postnatal depression scale, Depressive symptoms, Post-partum, Mothers, Nepal

## Abstract

**Background:**

Post-partum depression is a common complication of women after childbirth. The objective of this study was to determine the prevalence of and factors associated with depressive symptoms among post-partum mothers attending a child immunization clinic at a maternity hospital in Kathmandu, Nepal.

**Methods:**

This cross-sectional study was conducted among 346 post-partum mothers at six to ten weeks after delivery using systematic random sampling. Mothers were interviewed using a semi-structured questionnaire. The Edinburgh Postnatal Depression Scale (EPDS) was used to screen for depressive symptoms. Logistic regression analysis was used to calculate the association of post-partum depressive symptoms with socio-demographic and maternal factors.

**Results:**

The prevalence of post-partum depressive symptoms among mothers was 30%. Mothers aged 20 to 29 years were less likely to have depressive symptoms (adjusted odds ratio (aOR) = 0.40; 95% CI: 0.21-0.76) compared to older mothers. Similarly, mothers with a history of pregnancy-induced health problems were more likely to have depressive symptoms (aOR = 2.16; CI: 1.00-4.66) and subjective feelings of stress (aOR = 3.86; CI: 1.84-4.66) than mothers who did not.

**Conclusions:**

The number of post-partum mothers experiencing depressive symptoms was high; almost one-third of the participants reported having them. Pregnancy-induced health problems and subjective feelings of stress during pregnancy in the post-partum period were found to be associated with depressive symptoms among these women. Screening of depressive symptoms should be included in routine antenatal and postnatal care services for early identification and prevention.

## Background

Mental health problems are a major public health issue for women of reproductive age (15–44 years) in both high and low-income countries. About 7% of the global burden of diseases among women is contributed to mental health problems, especially among women of reproductive age [1]. The term post-partum depression (PPD) refers to a non-psychotic depressive state that begins in the post-partum period, after the child birth [2-4]. PPD is a mood disorder that can occur at any time during the first year after delivery [1,5]. Approximately 10-15% of women in the childbearing age experience this common complication of PPD [1]. PPD affects the health of not only the mother, but also of her children, especially mother-child bonding and the relationships among family members [6]. At present, PPD is not classified as a separate disease but diagnosed as an affective or mood disorders, according to the in Statistical Manual of Mental Disorders (DSM-IV) [7,8]. A meta-analysis of 59 studies from North America, Europe, Australia and Japan, found an overall prevalence rate of post-partum depressive symptoms of 13% [4]. A range of prevalence rates of post-partum depressive symptoms among post-partum women has also been found in developing countries, including India and Pakistan from 11 to 40% [9,10].

Earlier studies have found that risk factors for depressive symptoms are clustered into five major groups: biological, including changes in hormone levels and the age of mother; physical, including chronic health problems and antenatal depression; psychological, including prenatal anxiety, stress, lack of social support and stressful life; obstetrics/pediatrics, including unwanted pregnancy, history of loss of pregnancy and severely ill infants; and socio-cultural, including status of mother and poverty [6,11-15]. PPD has been associated with tragic outcomes, such as maternal suicide and infanticide [4]. Economic deprivations, gender of the infant, marital violence, smoking during pregnancy and hunger have also been associated with PPD [9,16].

A study conducted in the Dhanusha district of Nepal reported a number of factors associated with depressive symptoms among post-partum mothers, such as multiple births, caesarean section deliveries, serious perinatal health problems, illiteracy of the mother, higher parity, poor antenatal care, never having a son, having an illiterate husband, and lower maternal age [17]. Psychological violence during pregnancy by an intimate partner was strongly associated with PPD [18]. Low self-esteem and socio-economic status were significant predictors for depressive symptoms among post-partum mothers [19,20]. The prevalence of PPD symptoms was reported to be between 5.0 and 22.2% in Nepal, in different studies with various time frames [17,21]. The Maternal Mortality and Morbidity Survey (2009) identified suicide as a leading cause of death (16%) among women of reproductive age, with depression being the main cause for this [22]. Women having depressive symptoms during the postnatal period have a higher chance of self-harm than others in later years [23,24]. Also, depressive symptoms during the postnatal period are potential causes for neglect of the child and child abandonment. There are few studies conducted in Nepal on PPD symptoms, including some older studies [17,21,25,26]. However, the focus of those studies was limited to an aboriginal group in Nepal [25], or they had a small sample size [21]. Thus, there is the need for further studies to report the prevalence and associated factors of PPD symptoms in the country. Findings from such studies will be helpful in the design of prenatal and postnatal counseling and support to mothers, with special attention to vulnerable groups. Therefore, the objectives of this study are to: (1) calculate the prevalence of PPD symptoms among mothers; and (2) identify the factors associated with depressive symptoms among post-partum mothers.

## Methods

### Study setting

Paropakar Maternity and Women’s Hospital (PMWH) is the largest public hospital for maternity care in Nepal. This hospital receives a number of diverse women in age, ethnicity and educational status [27]. The PMWH runs a child immunization clinic everyday, which provides BCG, DPT, Polio and Measles vaccine to infants. The children attending this immunization clinic are generally accompanied by their mothers. Therefore, using this as the site for recruitment of post-partum mothers. We excluded mothers having chronic health problems, mothers with severely ill children and care takers other than post-partum mothers.

### Sample, tools and data collection

We conducted a cross-sectional study using a semi-structured questionnaire between August and September of 2012. Mothers who visited the child immunization clinic at a period of six to ten weeks after delivery were included in the sampling frame for this study. Sample size was calculated using the formula N = Z^2^p q/L^2^ = 1.96^2^*(0.08)*(0.92)/(0.03)^2^, taking Z = 1.96, p = 8% = 0.08 (assuming prevalence of PPD = 8%) [17], q = 1– p = (0.92), allowable error (L) = 3%, and level of significance =5%. Assuming a non-response rate of 10%, the sample size was estimated to be 346. A systematic random sampling technique was used to identify the name of mothers from the immunization register.

A self-administered version of the EPDS tool was used to screen the depressive symptoms among post-partum mothers. The EPDS tool has been validated and used in different cultural settings, including Nepal [3,21,28-30]. The EPDS tool is a ten-item self-reporting scale that measures the intensity of depressive symptoms experienced within the last 7 days. Each statement is rated on a scale from 0–3 (from “Yes, most of the time”, to “no, not at all”), resulting in a total possible score ranging from 0–30. We added pertinent socio-demographic characteristics and maternal factors to the questionnaire in order to collect information based on literature review [9,21,28]. The Nepali version of a questionnaire was pre-tested in Tribhuwan University Teaching Hospital in Kathmandu; the necessary amendments were made to make the questionnaire clearly understandable to mothers. Face-to-face interviews were conducted to collect maternal and socio-demographic information.

### Measurement of variables

#### Outcome variable

The outcome variable of the interest in this study was the occurrence of PPD symptoms based on EPDS screening criteria. We used an EPDS score ≥10 as a cut-off point to classify the depressive symptoms which has a sensitivity of 91% and specificity of 84% [31]. An earlier study has stated that the EPDS could convincingly rule out the depressive symptoms among post-partum mothers when a cut-off point of ≥10 was used [32].

### Explanatory variables

The explanatory variables investigated were both socio-demographic and maternal factors. Socio-demographic factors included age, ethnicity, occupation, education, and socio-economic status of the family. The Government of Nepal has classified ethnicity into six groups: Dalit, disadvantaged Janajati, disadvantaged non-Dalit Terai caste group, religious minorities, relatively advantaged Janajati and upper caste group [33,34]. In this study, ethnicity was further dichotomized into a relatively disadvantaged ethnic group and a relatively advantaged ethnic group for analysis. Similarly, educational status of parents was categorized as literate and illiterate. Socio-economic status of the family was categorized according to Kuppuswamy’s Scale (KS) [35]. The KS is comprised three variables; namely education, occupation and monthly income of the family head. Scores were given as education from 1 to 7; occupation from 1 to 10 and income from 1 (25 USD) to 12 (500 USD). A total score was further categorized as upper class (KS 26–29), middle class (KS 11–25) and lower class (KS ≤10) [35]. Maternal factors included family size, birth weight of children, type of delivery, pregnancy-induced health problems and subjective feelings of stress recalled during the last six months. Pregnancy-induced health problems included hypertension, headache and pregnancy complications while subjective feelings of stress included loss of interest and feeling, upset or bad during the pregnancy and post-partum period.

### Statistical analysis

Data cleaning and coding was done by the first author on the same day as data collection. Data were entered in EpiData version 3.1.1 (The EpiData Association, Odense, Denmark) and analysis was done using SPSS version 19 (SPSS, Inc., Chicago, IL).

The descriptive analysis of the socio-demographic characteristics and maternal factors of the study participants were reported as total number and percentages first. The prevalence of depressive symptoms among post-partum mothers was reported as percentages. Chi-square analysis was used to test the significance of associations between outcome and explanatory variables. The variables associated in bivariate analyses (p < 0.15) were included in backward stepwise logistic regression analyses.

### Ethical considerations

Ethical approval for this study was received from the Institutional Review Board of the Institute of Medicine, Maharajgunj Medical Campus, Tribhuwan University, Kathmandu, Nepal. The first author explained the research objectives and took verbal consent from the participants before starting the interview. The participants were ensured that confidentiality of the data will be strictly maintained and told about their right to withdraw from the interview at any time.

## Results

### Socio-demographic characteristics of participants

Among the 346 respondents interviewed, the mean age was 24.0 (SD 4.7) years and the majority of respondents (72.0%) were 20–29 years of age. More than 90% of mothers were housewives and 83.5% of the respondents were literate. Two in five respondents were from the disadvantaged ethnic group (40.2%). More than 60% of the respondents belonged to middle class families. About one in ten mothers (9.8%) had subjective feelings of stress in the last six months (Table 1).

**Table 1:** **Socio-demographic and maternal factors of the post-partum mothers**

**Characteristics**	**Number**	**Percent**
**Current age of mother**; mean:24 (SD 4.7) years		
<20 years	45	13.0
20-29 years	250	72.3
≥30 years	51	14.7
**Ethnicity**		
Disadvantaged ethnic group	139	40.2
Advantaged ethnic group	207	59.8
**Occupation of mother**		
Non housewife	29	8.4
Housewife	317	91.6
**Birth order**		
One child	220	63.6
More than one child	126	36.4
**Breastfeeding status of mothers**		
Exclusively breastfeeding	258	74.6
Non-exclusively breastfeeding	88	25.4
**Education status of mothers**		
Literate	289	83.5
Illiterate	57	16.5
**Husband’s educational status**		
Literate	314	90.8
Illiterate	32	9.2
**Reason for reported stress**		
Husbands behavior	18	5.2
Economic condition	328	94.8
**Socio-economic status of the family**		
Lower class (KS ≤10)	60	17.3
Middle class (KS 11–25)	222	64.2
Upper class (KS 26–29)	64	18.5
**Family size**		
≤5	272	78.6
>5	74	21.4
**Weight of the child**		
<2500 gram	27	11.2
≥2500 gram	214	88.6
**Delivery status**		
Normal delivery	262	75.7
Assisted delivery	84	24.3
**Pregnancy-induced health problems**		
Yes	31	9.0
No	315	91.0
**Subjective feelings of stress up to six months**		
Yes	34	9.8
No	312	90.2

### Prevalence of depressive symptoms among post-partum mothers

Out of 346 post-partum mothers, 105 (30.3%) had depressive symptoms (Table 2). Among the sub-groups, about half (47.1%) of mothers ≥30 years of age had depressive symptoms. Likewise, the prevalence of depressive symptoms in mothers whose husbands were illiterate, who had pregnancy-induced health problems and mothers from a lower socio-economic class were 48.0%, 45.2% and 41.7%, respectively. More than half of the respondents (58.8%) who had subjective feelings of stress also had depressive symptoms during post-partum period (Table 3).

**Table 2:** **Prevalence of post-partum depressive symptoms among mothers**

**Post-partum mother with depressive symptoms (EPDS score ≥10)**	**Number**	**Percent**
Yes	105	30.3
No	241	69.7

**Table 3:** **Factors associated post-partum depressive symptoms among mothers**

**Characteristics**	**EPDS score ≥10 (%)**	**EPDS score ≤9 (%)**	**Unadjusted odds ratio (95% CI)**		**Adjusted odds ratio (95%CI)**	
**Age of mother**				P = 0.019		P = 0.020
<20 years	14 (31.1)	31 (68.9)	0.58 (0.22-1.17)		0.49 (0.21-1.16)	
20-29 years	67 (26.8)	183 (73.2)	0.41 (0.22-0.76)		0.40 (0.21-0.76)^*^	
≥30 years ^#^	24 (47.1)	27 (52.9)	1.00		1.00	
**Education status of mother**				P = 0.140		P = 0.344
Literate	83 (28.7)	206 (71.3)	1.56 (0.86-2.81)		0.58 (0.19-1.77)	
Illiterate^#^	22 (38.6)	35 (61.4)	1.00		1.00	
**Husband’s educational status**				P = 0.036		P = 0.076
Literate	90 (28.6)	224 (71.4)	2.196 (1.05-4.58)		2.01 (0.93-4.38)	
Illiterate^#^	15 (46.8)	17 (53.1)	1.00		1.00	
**Reason for reported stress**				P = 0.002		P = 0.186
Husbands behavior	12 (66.7)	6 (33.3)	5.05 (1.84-13.86)		2.50 (0.64-9.71)	
Economic condition^#^	93(28.4)	235 (71.6)	1.00		1.00	
**Socio-economic status**				P = 0.111		P = 0.529
Lower class (KS ≤10)	25 (41.7)	35 (58.3)	1.97 (0.92-4.20)		1.57 (0.65-3.79)	
Middle class (KS 11–25)	63 (28.4)	159 (71.0)	1.09 (0.58-2.05)		1.07 (0.55-2.08)	
Upper class (KS 26–29)^#^	17 (26.6)	47 (73.4)	1.00		1.00	
**Pregnancy-induced health problems**				P = 0.064		P = 0.050
Yes	14 (45.2)	17 (54.8)	2.02 (0.96-4.28)		2.16(1.001-4.66)^*^	
No^#^	91 (28.9)	224 (71.9)	1.00		1.00	
**Subjective feelings of stress**				P < 0.001		P < 0.001
Yes	20 (58.8)	14 (41.2)	3.81 (1.84-7.89)		3.86 (1.84-8.09)^*^	
No^#^	85 (27.2)	227(72.8)	1.00		1.00	

^#^Reference category, ^*^statistically significant at p < 0.05.

### Factors associated with post-partum depressive symptoms

The current age of mothers, educational status of mothers and fathers, reasons for reported stress, socio-economic status of families, pregnancy-induced health problems and subjective feelings of stress in the last six months were all found to be associated with depressive symptoms among post-partum mothers in bivariate analyses. However, in the logistic regression analysis, only three variables were significantly associated with depressive symptoms. Mothers aged 20–29 years were less likely to report depressive symptoms (aOR = 0.40; 95% CI: 0.21-0.76). Similarly, mothers who had pregnancy induced problems (aOR = 2.16, 95% CI: 1.00-4.66) or were suffering from subjective feelings of stress (aOR = 3.86, 95% CI: 1.84-4.66) had a higher likelihood of having depressive symptoms (Table 3).

## Discussion

The present study showed that about one-third of mothers had depressive symptoms during post-partum period. Earlier studies reported prevalence of depressive symptoms varies 6 to 12% among women in the post-partum period using an EPDS score cut-off of ≥ 12 [29,30]. A recent cohort study conducted in two hospitals of Nepal, in Dhading and Kathmandu, using an EPDS score ≥13 as a cut-off point, found that 22.2% of post-partum mothers had depressive symptoms [21]. In other developing countries including India and Pakistan, the prevalence of depressive symptoms among post-partum mothers have been reported as 11 to 40% [30]. The high prevalence of depressive symptoms reported in this study might be because of lower cut-off point of EPDS score (≥10) compared to previous studies (≥12) [16,29].

The current study found mothers 20–29 years of age were less likely to have depressive symptoms compared to the mothers of the oldest age group. In contrast, earlier studies in Turkey and Canada reported that mothers aged less than 25 years were more likely to report depressive symptoms [36-38]. However, some other studies have found no differences in depressive symptoms by age [39-41]. Women between 20–29 years of age may be more aware of pregnancy and pregnancy outcomes, and thus develop confidence to cope with it; in this age group, women in Nepal usually have a parity greater than two [42]. Additionally, this reduces the risk of pregnancy complications further, so these mothers might be at lower risk for depressive symptoms compared to mothers of other ages.

We found that pregnancy-induced health problems and subjective feelings of stress during the last six months were significantly associated with depressive symptoms. Stressful situations during pregnancy, such as the pain response to vaginal birth, have been reported to increase the chances of depressive symptoms among post-partum mothers [43]. The earlier study conducted in Nepal also showed that stressful life events were associated with depressive symptoms [16]. Depressive symptoms were most common among post-partum mothers who had hyperemesis or premature contractions during pregnancy [44]. Stressful events such as maternity blues on the seventh day after delivery, obsessive preoccupation with cleaning, and judgment that the baby is crying excessively at first were also factors significantly associated with depressive symptoms among post-partum mothers [45]. A review also reported that poor marital adjustment, recent life stressors, and ante-partum depression were strong predictors of PPD symptoms [46].

We did not find any association of depressive symptoms with socio-demographic factors except for the age of mothers. O’Hara and Swain (1996) found that poor marital relationships and low social support were significantly associated with depressive symptoms [4]. A previous study in Nepal showed significant association of depressive symptoms with low income, maternal occupation and low social status [17].

The screening of depressive symptoms among post-partum mothers with pregnancy-induced health problems [47,48] and/or subjective feelings of stress are the programmatic implications of this study. Our study has a number of limitations. The EPDS tool used in this study is only a symptomatic assessment for depressive symptoms without a clinical diagnosis. We cannot rule out the possibility of recall bias of the respondents reported information. Having selected an immunization clinic as our setting, the findings from this study might not be generalizable to the community setting. While there is growing evidence that smoking and alcohol affect maternal and child health, we did not collect data on these variables, which could be seen as a major limitation of this study. Further research in this area should explore the role of pregnancy complications, smoking and alcohol use on depressive symptoms among mothers, and be carried out among a representative population, using a robust study design.

## Conclusions

About one-third of post-partum mothers attending the immunization clinic at a maternity hospital in Nepal had reported depressive symptoms. Pregnancy-induced health problems and stress during pregnancy were associated with occurrence of PPD symptoms. The screening of depressive symptoms among post-partum mothers who have pregnancy-induced health problems and subjective feelings of stress during pregnancy should be included in routine maternity care services.
